# Retention Time Variability as a Mechanism for Animal Mediated Long-Distance Dispersal

**DOI:** 10.1371/journal.pone.0028447

**Published:** 2011-12-14

**Authors:** Vishwesha Guttal, Frederic Bartumeus, Gregg Hartvigsen, Andrew L. Nevai

**Affiliations:** 1 Department of Ecology and Evolutionary Biology, Princeton University, Princeton, New Jersey, United States of America; 2 Centre for Ecological Sciences, Indian Institute of Science, Bangalore, India; 3 Center for Advanced Studies of Blanes, CEAB-CSIC, Blanes, Spain; 4 Department of Biology, SUNY Geneseo, College Circle, Geneseo, New York, United States of America; 5 Department of Mathematics, University of Central Florida, Orlando, Florida, United States of America; Netherlands Institute of Ecology, Netherlands

## Abstract

Long-distance dispersal (LDD) events, although rare for most plant species, can strongly influence population and community dynamics. Animals function as a key biotic vector of seeds and thus, a mechanistic and quantitative understanding of how individual animal behaviors scale to dispersal patterns at different spatial scales is a question of critical importance from both basic and applied perspectives. Using a diffusion-theory based analytical approach for a wide range of animal movement and seed transportation patterns, we show that the scale (a measure of local dispersal) of the seed dispersal kernel increases with the organisms' rate of movement and mean seed retention time. We reveal that variations in seed retention time is a key determinant of various measures of LDD such as kurtosis (or shape) of the kernel, thinkness of tails and the absolute number of seeds falling beyond a threshold distance. Using empirical data sets of frugivores, we illustrate the importance of variability in retention times for predicting the key disperser species that influence LDD. Our study makes testable predictions linking animal movement behaviors and gut retention times to dispersal patterns and, more generally, highlights the potential importance of animal behavioral variability for the LDD of seeds.

## Introduction

Dispersal is the unidirectional movement of an organism, or its reproductive unit (e.g., seeds), away from the place of its origin [1]. In many plant species, a major portion of dispersal events happen close to the parent plant and this short ranged dispersal is an important process that influences both local and larger scale dynamics of the population. It has been suggested that the relatively infrequent but long-distance dispersal (LDD) can also significantly impact larger spatial scale processes such as population abundance, spread, and coexistence with other species [1]–[3]. Due to the role played in determining ecological patterns at higher levels of organization, understanding factors which drive both short-distance and LDD events can provide useful insights into biodiversity management and conservation biology in the context of exotic species invasion, spread of diseases and landscape fragmentation in an increasingly changing world due to anthropogenic influences [4]–[6].

While short-distance dispersal has been well studied for a long time, it is only relatively recently that the significance of the basic as well as applied aspects of LDD in ecology [1] and epidemiology [7] have been well recognized. This has led to a surge in empirical and theoretical studies to device quantitative measures of LDD events. Dispersal patterns are often quantified using dispersal distance kernels/curves, which are functions that describe the probability density of a dispersal unit being deposited at a certain distance from the parent source. Local or short-distance dispersal, *i.e.*, the typical distance within which most of the seeds fall, is often determined by the scale, or standard deviation, of the curve together with its mean [8]. Long-distance dispersal has been quantified by a number of measures such as kurtosis [8]–[10], thickness (or fatness) of tails of dispersal kernels [2] and/or absolute measures such as number of seeds falling beyond a threshold distance [11]. The kurtosis, or the shape, of the kernel measures the distribution of the probability density at both the peak and tails of the kernel [12].

Despite certain limitations involved in using kurtosis as an unambiguous measure of LDD [3], it is widely employed in theoretical [14], simulational [8] as well as empirical studies ([9], [10]). We further note that different definitions of fat-tails are employed in the literature. For example, one approach requires that the tail of the kernel decay at a rate slower than the negative exponential curve [11]. Alternatively, some authors have employed a less restrictive definition that in a fat-tailed kernel, the tail may decay at a rate slower than a Gaussian tail [15]. Theoretical results show that if the tail of the dispersal curve decays like a Gaussian or negative exponential, then the population advances at a constant speed [2], [16], [17]. In contrast, curves with fatter tails can lead to an accelerating rate of invasion of the habitat thus having a large scale and disproportionate impact on population structure [2], [4].

Quantifying LDD in the field, however, is a challenging task owing to its infrequent nature which results in lack of data and reliable statistics [18]–[20]. Therefore it is critical to reveal processes which are key to the formation of a kernel with given statistical properties. More specifically, to gain predictive power on dispersal patterns, one must identify not only the LDD events but also dispersal agents and mechanisms driving these rare events [1], [11]. Wind is a major disperser of seeds and a number of studies based on analytical models, numerical simulations and field data analysis have shown that correlated wind structures, incidence upward drift and seed release during gusts can all drive LDD [21]–[23]. Besides wind, animals form a major vector of dispersal units. Field studies and detailed simulation models have shown that the behavior of dispersers [24]–[32], together with the habitat characteristics and landscape heterogeneity [8] significantly affect dispersal patterns and, in particular, different measures of LDD. With growing interest in animal mediated dispersal there is an increasing need to develop simple and broadly applicable analytical models that can present clear links between measurable aspects of animal behavioral ecology and dispersal events, including LDD. Such a theoretical model can not only provide a better comprehension of the underlying processes influencing dispersal patterns but may also offer useful insights into conservation strategies by identifying key dispersal vectors.

Here, we employ an analytical approach based on a diffusion-theory to link animal movement behavior and seed transportation dynamics to dispersal patterns at different spatial scales (*i.e.*, local and long range dispersal) in one and two spatial dimensions. In particular, we show how the scale (a measure of local dispersal), the kurtosis, the thickness of tail of dispersal kernels (measures of LDD) are determined by animal movement and gut retention time patterns. We also determine how an absolute measure of LDD, defined as the number of seeds falling beyond a threshold distance, is influenced by seed retention time patterns. We show the generality of our results by considering a variety of movement patterns (*e.g.*, diffusive, drift, correlated random walks and home-ranges) and retention time patterns (*e.g.*, passage through the gut, adhesion to the body) likely to be exhibited by animals. We analyze gut-passage time data from the published literature and bird species of a Mediterranean ecosystem and make predictions on the key long-distance dispersers. Finally, we discuss ecological implications of our results, limitations of our study and possible future work.

## Methods and Results

During the process of animal mediated dispersal the combined effects of two basic processes, the movement pattern of foraging animals and the method of seed transportation, determine when and where seeds will be released. In this section we describe a simple model to determine how these processes contribute to the construction of a seed dispersal kernel. Since the mathematical framework is general, it can be applied to the dispersal of other units as well (such as pathogens and other micro-organisms).

We assume that seed dispersal processes occur in a spatial domain 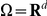 (where 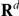 represents 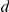 spatial dimensions) and that all seeds originate from a single source, 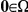. In calculating the eventual seed dispersal pattern, we assume that animals vary probabilistically in both their movement pattern and seed retention time. Let 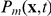 be the probability density that an animal will be at position 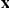 after 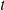 units of time since collecting a seed, and let 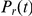 be the probability density that an animal retains the seed for 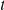 units of time. Then the probability density that an animal will release a seed at location 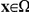 is obtained by adding the contributions of different dispersal events generated by all probable combinations of animal displacement and seed retention time [8], [26], [33], [34], *i.e.*,
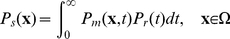
(1)We refer to 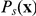 as the *seed dispersal kernel* (see Table 1 for a summary of model parameters).

**Table 1:** Summary of model parameters.

Quantity	Description	Dimensions
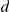	number of spatial dimensions	-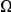	spatial domain	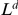
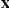	location in 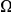	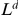
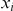	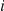 th coordinate of 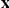	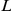
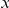	location in 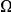 (when 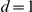 )	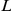
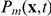	animal movement pattern	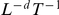
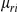	mean animal displacement	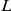
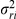	variance of animal displacement	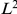
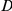	diffusion constant	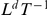
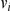	velocity	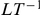
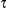	correlation time	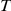
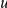	speed	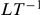
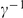	average return-time to nesting site in the home-range model	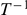
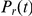	seed retention time	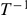
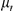	mean seed retention time	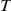
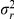	variance of seed retention time	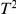
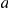	shape parameter of Gamma distribution	-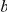	scale parameter of Gamma distribution	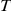
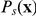	seed dispersal kernel	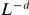
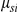	mean seed displacement	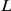
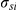	standard deviation of seed displacement	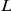
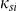	excess kurtosis of seed dispersal kernel	-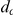	threshold dispersal distance	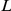
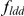	proportion of seeds falling beyond a threshold dispersal distance	-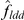	normalized 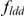 such that, for each fixed 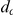 , 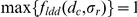	-

Note that 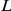 = length, 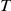 = time. A subscript of 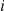 indicates “in the 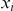-direction” (when 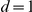 it is omitted).

### Retention time variability can lead to leptokurtic dispersal

Although most animals can move in complex ways within their habitats [35], we will begin our analysis by assuming that animals move randomly in a one-dimensional environment 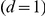 that is both homogeneous and isotropic so that their movement pattern is independent of position and direction. In addition, we assume that individuals do not interact with each other in ways that can alter their movement pattern between the time of seed collection and release. Under these simplistic assumptions, which have been widely employed in the literature to obtain generic principles of movement ecology [17], [35], we recover a familiar form of movement pattern, diffusion. Here, the probability density that an animal will be at location 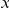 after 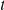 units of time is [17]:
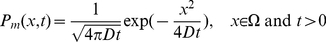
(2)Here, the diffusion constant 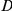 is a measure of an organism's rate of movement or the population's *spreading rate*.

Let 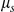, 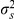, and 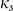 denote the mean, variance, and (excess) kurtosis, respectively, of the seed dispersal kernel 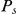 (formal definitions appear in Text S1). Based on Eqs (1) and (2), we show that (see Text S2)
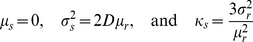
(3)The standard deviation or *scale* (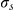) is a commonly-used measure of relatively short, or local, dispersal distance. Here, it is seen to increase with spreading rate 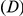 and mean seed retention time 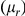. The kurtosis or *shape* 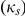 is a frequently used measure of long-distance dispersal [8], [10], [14]. A positive (or negative) kurtosis or shape indicates that events at the peak and tail together occur more (or less) frequently than a Gaussian model would predict. As can be seen in Eq (3), the kurtosis of the seed dispersal kernel is non-negative, positively related to variation in seed retention time 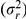, and inversely related to mean seed retention time 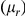. In contrast, it is unaffected by the spreading rate 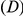. In other words, variations in the seed retention time is a key generator of leptokurtic seed dispersal kernels. We also emphasize the generality of these results by noting that the expressions in Eq (3) do not depend on the explicit form of the retention time distribution (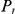), but only on the summary statistics (mean and variance) of 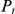.

### Retention time variability can lead to power-law seed dispersal kernel

Here, we obtain a sample seed dispersal kernel generated by a diffusively moving population of organisms. To do so, we need to assume a form for the seed retention time distribution (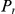; see Eq (1)).

#### Seed retention time distributions for endozoochory and epizoochory

Endozoochory (passage through the gut) is a widespread form of seed transportation [36], [37], and among animals which disperse seeds in this manner, perhaps the most commonly studied are avian and mammalian frugivores (*i.e.*, consumers of fleshy fruits). Hence, as a starting point, we assume that animals disperse seeds via endozoochory, and following the empirical work of [26], we assume that seed retention times obey a Gamma distribution:
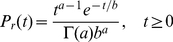
(4)where 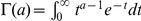. We remind the reader that a Gamma distribution can reproduce many different kinds of one-sided probability distributions, and moreover that its parameters 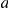 and 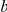 can be written in terms of its mean (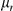) and variance (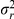) as follows
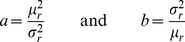
(5)Observe that 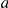 and 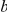 are both non-negative, and they respond in opposite ways to increases in 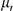 and 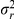.

It is worth remarking that for epizoochory, in which the transportation of seeds is determined by the purely physical process of adhesion, a seed may be released when the adhesive forces become relatively weak, as occurs when an animal's fur slides past an object. If the rate of occurrence of such an event is constant in time, then the probability that an animal will carry a seed for 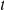 units of time will follow an exponential distribution, which is itself a Gamma distribution with 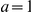. Furthermore, we note that gamma distribution is often considered a realistic choice for representing survival/waiting times that could be overdispersed or having large coefficients of variation [38].

#### Power-law seed dispersal kernel

We now substitute Eqs (2) and (4) into (1) and then integrate to obtain an expression for the seed dispersal kernel
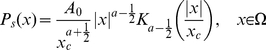
(6)Here, 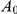 is a positive constant (depending only on 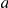), 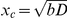, and 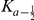 is a modified Bessel function of the second kind [39]. The asymptotic formula 
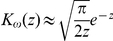
 for 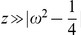 [39] allows us to approximate the seed dispersal kernel at large distances by
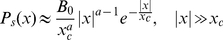
(7)where 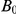 is a positive constant (depending only on 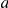). In view of this approximation, we see that 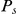 (which decays with distance 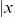 as 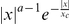) has a fatter tail than a Gaussian kernel (which decays as 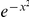). It is also important to note that 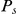 has a power-law behavior for shorter dispersal distances (from Eq (6)). For these reasons, we say that 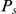 is a *power-law kernel with an exponential cut-off*. We note similar redistribution kernels have also been derived in the context of heterogeneous population structures and animal movement [15], [33].

**Figure 1:**
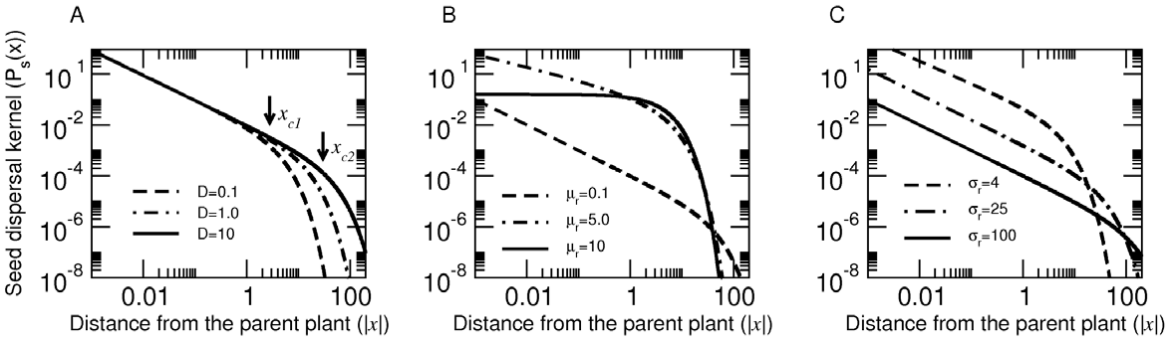
The seed dispersal kernel 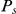 of Eq(6) as a function of movement and retention times. For different values of (A) Organism's spreading rate (or diffusion constant 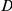), (B) mean seed retention time (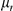) and (C) variation in seed retention time (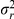). In (A), 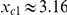 and 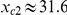 denote the cut-off distance of the power-law behavior for 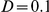 and 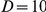 units. Parameters for (A) 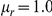 and 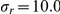; (B) 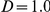 and 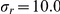; (C) 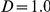 and 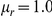.

In Fig. 1 we explicitly demonstrate the power-law nature of 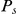 on spatial scales where the log-log plot exhibits a linear relationship. It is useful to refer to 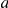 (in 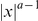) as the *scaling exponent* which can be estimated by the slope of the linear portion of the log-log 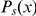 plot. A larger scaling exponent (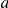) leads to slower decay of the dispersal kernel with distance; when 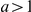, the power-law part grows with distance but is eventually overtaken by the exponential decay. Next, we define the *cut-off distance* (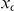) as a measure of the spatial scale at which the kernel begins to deviate from the power-law towards exponential decay; the larger the 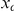 the farther the distance at which this deviation occurs.

We now establish links between parameters of the seed dispersal kernel and the two key behaviors (*i.e.*, movement and gut retention times) of the dispersing agent. An increase in the spreading rate of the disperser 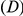 will increase the cut-off distance 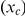, but the exponent of the power-law (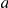) remains unaffected. In Fig. 1 A we explicitly illustrate that when 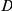 increases from 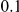 to 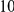 units, the deviation from power-law shifts from 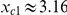 to 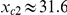; and the slope of the linear portion of the log-log plot (and hence the scaling exponent 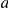) remains the same for different 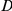. Furthermore, observe that an increase in the mean seed retention time 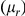 results in an increased power-law exponent (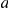); however, it reduces 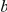 and hence 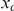 leading to a deviation from power-law at relatively shorter distances (Fig. 1 B). In contrast, an increase in seed retention time variability 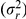 increases the cut-off distance (Fig. 1 C).

#### Kurtosis and thickness of tail of the kernel

We remind the reader that widely used quantities of scale and kurtosis of the seed shadow continue to obey Eq (3). In particular, the measure of kurtosis suggests that the larger the variation in seed retention time, the higher will be long-distance dispersal events in comparison to a Gaussian-like tail. However, the effectiveness of kurtosis as a measure of long-distance dispersal is sometimes questioned [13] because it measures both peakedness and heaviness in tails of a probability distribution [12]. Therefore, it is theoretically possible to construct dispersal kernels where an increased kurtosis may occur only due to peakedness but having no long range dispersal.

To investigate how an increased kurtosis affects the strength of probability distribution at the tail for animal mediated dispersal kernel (Eq (7)), we perform the following analysis. We begin by considering zero variation in retention times (*i.e.*, 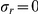) that results in a kurtosis of seed dispersal kernel to be 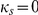 (from Eq (3)). In this special case every animal retains a seed for exactly 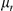 units of time before releasing it. Substituting 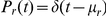 (where 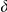 is the Dirac-delta function) and Eq (2) into Eq (1), we obtain
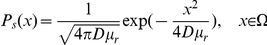
which indeed is a Gaussian kernel. This, in conjunction with the power-law dispersal with an exponential decay of Eqs (6–7), shows that a non-zero retention time variability, and consequently a non-zero kurtosis, does indeed lead to LDD events, as measured by thickness of kernels.

**Figure 2:**
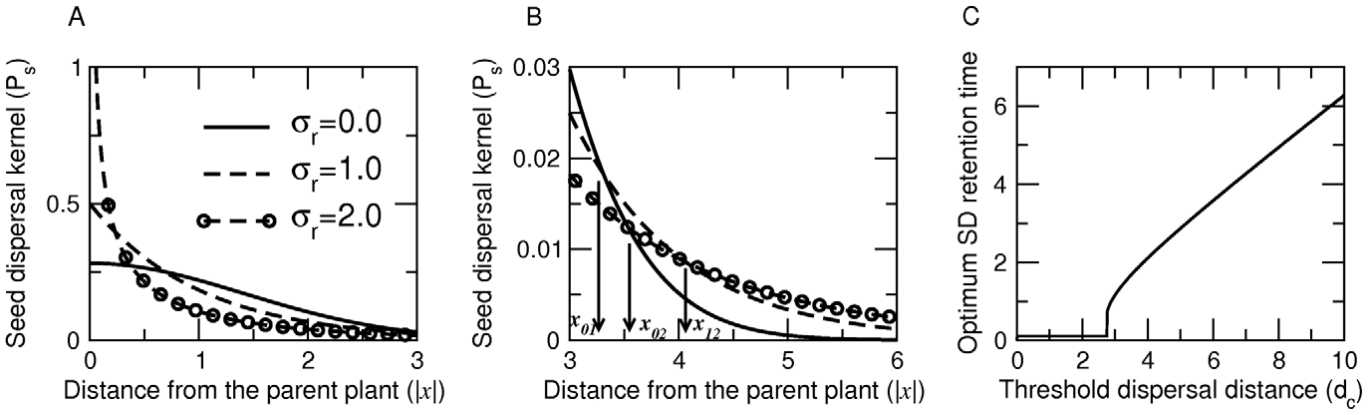
Variation in seed retention time (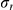) that maximizes LDD events. (A) 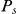 for different values of 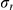. (B) 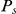 at large distances. The symbol 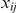 (*e.g.*, 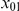) indicates the distance at which a seed dispersal kernel with 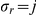 (*e.g.*, 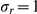) begins to have more frequent long-distance dispersal events than a seed dispersal kernel with 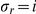 (*e.g.*, 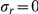). As 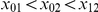, a larger variability in retention time (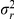), thus a larger kurtosis of seed dispersal kernel, leads to fatter seed dispersal tails beyond a threshold distance that increases with 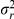. (C) Optimum value of seed retention time that maximizes absolute LDD (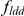) (defined as the proportion of seeds falling beyond a threshold dispersal distance 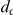; also see Text S9). Here we employed two dimensional diffusion with 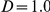 and 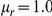.

We determine the implications of increasing variations in retention time (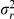) on the tail of seed dispersal kernel in more detail. We find that, (i) as 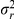 increases, the probability of seed deposition is higher than the Gaussian kernel but only beyond a critical distance, denoted by 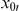 (Fig. 2 A–B). Note that the symbol 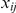 (*e.g.*, 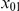) indicates the distance at which a seed dispersal kernel with 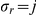 (*e.g.*, 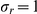) begins to have more frequent LDD events than a seed dispersal kernel with 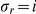 (*e.g.*, 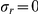). Our computations further reveal that this distance (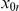) increases with an increase in 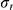 (see Fig. 2 A–B), and consequently with the kurtosis of the seed dispersal kernel (Eq (3)). To give a simple numerical example, when 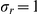 the seed dispersal kernel will have more dispersal events than a Gaussian kernel (generated by 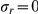) would suggest beyond 3.3 units of distance 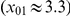. For a higher value of 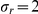 (and hence higher kurtosis in the seed dispersal kernel) we have 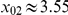 units. In other words, larger variations in seed retention time does indeed lead to higher frequency of dispersal events, than a Gaussian kernel would predict, beyond a threshold distance.

### Retention time variability and an absolute measure of LDD

LDD has also been quantified based on absolute measures ([11]; see Text S9) such as the number of seeds falling beyond a certain threshold distance (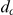). It has been shown that larger the organismal rate of movement and mean seed retention times, the larger will be this absolute LDD [11]. Here, we consider how this is influenced by retention time variability.

We compute this absolute measure of LDD for a range of 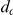 and 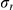. For each threshold distance (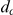), we find that there is an optimum variation in seed retention time 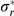 at which the absolute LDD is maximum (Text S9). We then plot 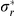 as a function of the threshold distance (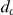), which forms a pitchfork-like pattern as shown in Fig. 2 C. When the threshold distance is small, *i.e.*, long-distance dispersal events are not important, there is no need for variation in the retention time. As the threshold dispersal distance increases, the optimal variation in seed retention time also increases.

### Generality of results

In this section we test the generality of our results by relaxing various model assumptions. We begin by examining different animal movement patterns 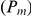.

#### Animal movement patterns (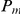)

Animal movement patterns can exhibit a variety of macroscopic properties. Depending on how directional correlations build up, movement patterns can exhibit diffusive, super/sub-diffusive, and/or advective properties over a wide range of spatio-temporal scales [40]. By means of different random walk models, we can explore the generality of our main results.

**Figure 3:**
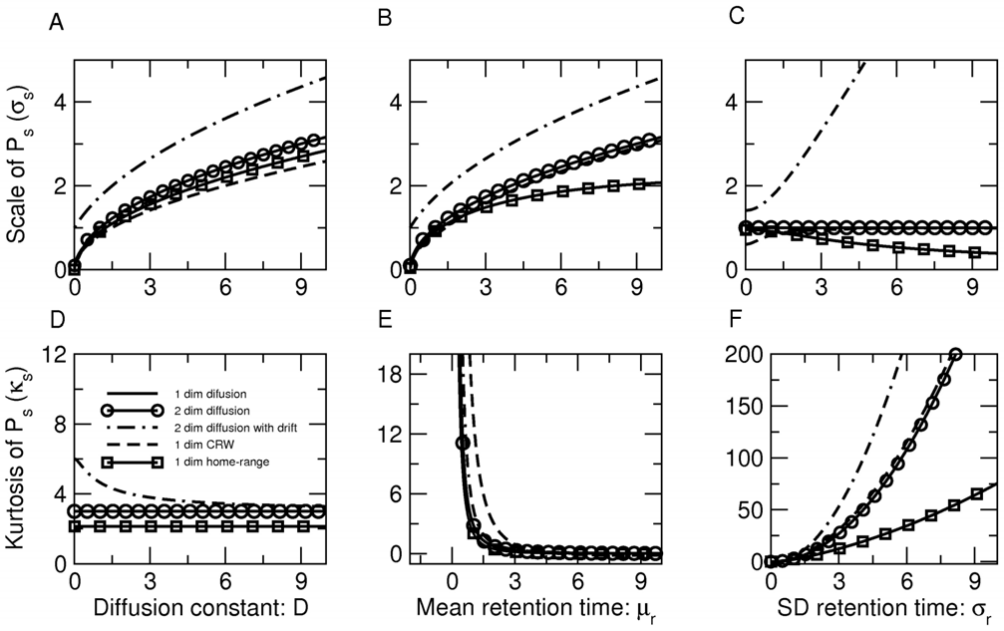
Scale and kurtosis of 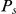 for four different patterns of animal movement. Top row of the panel shows scale as a function of the diffusion constant in (A), mean seed retention time in (B), and standard deviation (SD) in seed retention time in (C). In (C) we also demonstrate that in the model with drift, scale increases with SD in retention time and thus differs notably from other movement models. Bottom row (D)–(F) shows kurtosis as a function of the same parameters. Although the qualitative features of kurtosis remain the same across different movement models, it is always larger for the model with drift. For 2 dimensional models, we have plotted scale and kurtosis along one dimension. Parameters for (A) and (D): 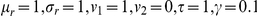; for (B) and (E): 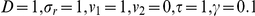; for (C) and (F): 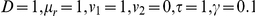. See Table 1 for a description of parameters.

To begin, consider diffusive movement in a two-dimensional environment 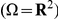 and let 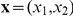. As shown in Text S3, the mean, scale, and shape of the seed dispersal kernel (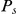) along each of the two dimensions (say for the 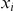-axis, we denote them by 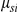, 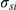, and 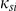) all obey the same formulas as their counterparts in Eq (3) (see Fig. 3). In addition, the full kernel 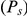 continues to possess a power-law decay with an exponential cut-off.

Next, suppose that the diffusive motion of animals in two spatial dimensions possesses a drift component in some particular direction. A drift can result for a variety of reasons, including the presence of wind or water, an animal's migratory behavior, or the influence of an elevational gradient [17]. Using the theory of drift-diffusion (also known as advection-diffusion) equations [17], we show in Text S4 that the summary statistics of the seed dispersal kernel (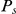) are now given by
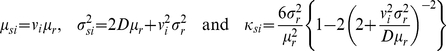
The mean seed displacement 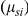 is proportional to the velocity 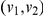 of the advective motion, and the scale 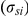 increases with both the mean and variation in seed retention time 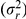. Both of these results differ qualitatively from the pure diffusion models. Since the kurtosis or shape is larger than the corresponding value in Eq (3), the likelihood of long-distance dispersal is increased when animals have a drift component to their movement (Fig. 3). We also show that the full seed dispersal kernel 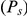 still possesses a power-law structure with an exponential cut-off (Text S4).

Suppose now that animals follow correlated random walks (CRW; [35]). A CRW incorporates directional persistence into diffusive motion, reflecting the tendency of randomly moving animals to keep moving in the same direction over a short time scale 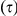; this is in contrast to drift-diffusion movement where a constant directional bias exists at all timescales (*e.g.*, downstream of a river). Due to these directional correlations, the wave-front of the animal movement pattern moves at a finite speed, eliminating a dubious feature of diffusive motion in which the wave-front moves infinitely fast [17]. For this movement pattern, a closed form for the seed dispersal kernel 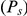 cannot be obtained. Yet, for larger time scales (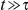) the directional correlations decay, resulting in what is effectively diffusive motion [35], [40]. Therefore we expect the qualitative features of the seed dispersal kernel obtained for diffusive motion (*i.e.*, LDD generated through variations in seed retention time), to continue to hold for this movement pattern as well. To support our claim, we show in Text S5 how the scale 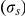 and shape 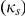 of 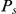 are determined by the effective spreading rate 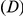, the mean seed retention time 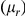, and seed retention time variability 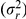 (also see Fig. 3). We find that as the correlations reduce (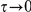), the summary statistics of 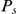 reduce to their counterparts in Eq (3) for diffusive motion.

In order to avoid competition for resources and/or to reduce predation risks, many animal species possess home-ranges leading to a bounded movement pattern [41]. To model this we assume that, in addition to randomness in motion, animals have a preference to return to a fixed point in space. In Text S6 we show that the features of short-distance dispersal of seeds (*i.e.*, scale) differs qualitatively in comparison to the results of the previous random walk models: it saturates asymptotically to a non-zero constant as mean seed retention time 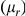 increases and declines to zero as seed retention time variability 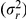 increases. However, the qualitative features of kurtosis remain unaffected (see Fig. 3).

Finally, we consider another extreme in which the animal movement pattern possesses super-diffusive properties over a wide range of scales, *e.g.*, Lévy flights [40]. In Text S7 we utilize a recent model of animal movement in which a power-law animal displacement kernel originates in a statistically structured population [15]. We show that a power-law movement pattern alone can generate LDD of seeds, as one would expect intuitively, even when there is no variability in seed retention time (Text S8).

#### Seed retention times (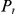)

We now consider the role of the seed retention time distribution (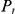). Observe from Eq (3) (and its derivation in Text S2) that the scale 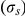 and shape 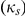 of 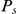 depend only on the mean 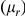 and variance 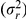 of 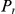, and not on its particular form. This could imply that details associated with specific mechanisms of seed retention times such as endozoochory, epizoochory, and regurgitation of seeds [42] may be less important in driving LDD, as measured by kurtosis of 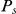, than the mean 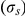 and variations 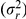 in seed retention times generated by these processes. We note that the Gamma distribution has specific features that can potentially make our results less general; it has a power-law with an exponential cut-off (see the term 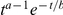 in Eq (4)) and it allows for the occurrence of arbitrarily large values of retention times. In Text S9, based on techniques of ref [15], we argue that a power-law in the seed dispersal kernel (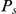) appears, albeit for a reduced range of spatial scales, even for seed retention time distributions that do not have these characteristics.

### Predicting key LDD vectors from empirical data

We consider two empirical data sets for endozoochorial seed retention times (or gut-passage times) in frugivores and use them to predict key vectors responsible for LDD, as measured by kurtosis [10]. Our predictions based on gut-passage time variability (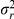) identify some vectors as being potentially responsible for LDD, despite the fact that their mean seed retention times 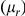 are not among the highest.

Our first data set (Table 2) contains mean 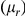 and standard deviation 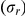 gut-passage times for a variety of plant-frugivorous interactions appearing in the published literature [29], [43], [44]. To compute seed dispersal kernel kurtosis values for each plant-animal interaction, we assume that birds move diffusively in two dimensions while foraging fruits, apply Eq (3) to predict the kurtosis 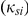 of the seed dispersal kernel along each of the two spatial dimensions, and then obtain the total kurtosis 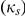 by summing 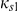 and 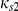. Note that, in the absence of movement data for the birds considered in this study, we make a simplistic assumption that they move diffusively; however, based on our analysis in the section Generality of results, we expect that the qualitative features of the following analysis will continue to hold.

**Table 2:** Seed retention time data from the published literature.

Bird species	Plant species	# of seeds fed	Gut passage time	Kurtosis ( 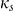 )	Rank 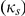	Reference
*Casuarius casuarius*	*Aceratium sericoleopsis*	405	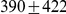	7.025	1	[29]
*Casuarius casuarius*	*Cryptocarya pleurosperma*	55	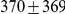	5.967	2	[29]
*Casuarius casuarius*	*Davidsonia pruriens*	79	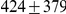	4.793	3	[29]
*Casuarius casuarius*	*Elaeocarpus grandis*	238	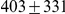	4.047	4	[29]
*Casuarius casuarius*	*Ficus crassipes*	5730	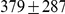	3.440	5	[29]
*Casuarius casuarius*	*Normanbya normanbyi*	100	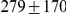	2.227	6	[29]
*Casuarius casuarius*	*Acmena divaricata*	4	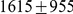	2.098	7	[29]
*Casuarius casuarius*	*Endiandra longipedicillata*	127	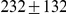	1.942	8	[29]
*Casuarius casuarius*	*Elaeocarpus largiflorens*	341	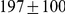	1.546	9	[29]
*Casuarius casuarius*	*Peripentadenia mearsii*	333	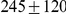	1.439	10	[29]
*Musophaga johnstoni*	*Syzygium parvifolium*	46	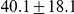	1.222	11	[43]
*Casuarius casuarius*	*Endiandra impressicosta*	125	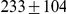	1.195	12	[29]
*Musophaga johnstoni*	*Psychotria mahonii*	4	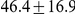	0.795	13	[43]
*Musophaga johnstoni*	*Maesa lanceolata*	6	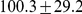	0.508	14	[43]
*Musophaga johnstoni*	*Ekebergia capensis*	9	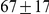	0.386	15	[43]
*Ceratogymna cylindricus*	*Enantia chlorantha*	6	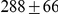	0.315	16	[44]
*Musophaga johnstoni*	*Balthasarea schliebeni*	3	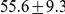	0.167	17	[43]
*Ceratogymna cylindricus*	*Maesopsis eminii*	3	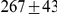	0.156	18	[44]
*Musophaga johnstoni*	*Ilex mitis*	4	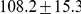	0.119	19	[43]
*Ceratogymna atrata*	*Cleistopholis patens*	27	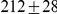	0.104	20	[44]
*Ceratogymna cylindricus*	*Strombosia scheffleri*	19	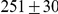	0.086	21	[44]
*Ceratogymna atrata*	*Xylopia hypolampra*	26	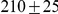	0.085	22	[44]
*Ceratogymna cylindricus*	*Ficus sp.*	23	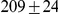	0.079	23	[44]
*Ceratogymna atrata*	*Staudtia stipitata*	30	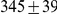	0.076	24	[44]
*Ceratogymna atrata*	*Rauwolfia macrophylla*	19	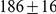	0.044	25	[44]
*Ceratogymna cylindricus*	*Lannea sp.*	20	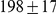	0.044	26	[44]
*Ceratogymna atrata*	*Maesopsis eminii*	17	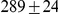	0.041	27	[44]
*Ceratogymna cylindricus*	*Xylopia hypolampra*	38	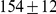	0.036	28	[44]
*Ceratogymna cylindricus*	*Staudtia stipitata*	22	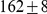	0.015	29	[44]

All plant-animal interactions are ranked according to their predicted kurtosis, with a higher kurtosis indicating that the interaction is more likely to result in the long-distance dispersal of seeds belonging to the plant species. Gut passage times are expressed as mean (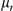) 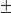 SD (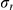) (in minutes). Kurtoses are predicted values based on assumed two-dimensional random movement (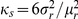).

First, we find that the same bird species (*e.g., Casuarius casuarius*) can exhibit large differences in its seed dispersal characteristics (as measured by kurtosis) depending on the type of the seed it consumes and the associated fruit and seed digestive processing. Second, for several plant species there exist multiple frugivores that consume their seeds and are responsible for its dispersal. Based on our kurtosis calculations we predict the relative importance of vectors responsible for LDD. For example, bird species *C. cylindricus* is likely to fair better as a long-distance disperser of plant species *Maesopsis eminii* than *C. atrata* (ranked 18 and 27, respectively); a prediction based on mean seed retention times alone could not have made such a distinction.

Our second empirical gut-passage time data is obtained from P. Jordano (Estación Biológica de Doñana, CSIC), and includes species representative of the avian frugivore fauna of Mediterranean ecosystems. The bird species listed in Table 3 are primarily frugivorous except *M. striata* (ranked 8), *S. torquata* (10), *F. hipoleuca* (12), and *S. undata* (13) all of which are primarily insectivorous but do disperse seeds occasionally. In dietary experiments, a solution of barium sulphate (an inert tracer that is not digested by birds) was administered, the time of first appearance of the marker in faeces and/or regurgitated seed(s) was noted, and the bird released (Jordano *et al*, unpublished). The inert tracer technique produces mean and standard deviation gut-passage time data without the influence of seed size, texture, laxative effects of pulp, etc. Therefore the kurtosis can be directly compared across different disperser species to predict the most effective LDD vectors for plants in this ecosystem.

**Table 3:** Retention time of an inert tracer (barium sulphate) in various Mediterranean bird species.

Bird species	# of trials	Gut passage time	Kurtosis ( 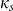 )	Rank 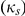
*Sylvia borin*	37	79.0 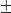 49.0	2.310	1
*Sylvia melanocephala*	59	33.0 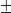 19.8	2.164	2
*Erithacus rubecula*	38	40.7 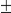 22.7	1.876	3
*Turdus merula*	7	59.1 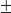 31.2	1.674	4
*Sylvia atricapilla*	37	36.51 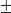 16.5	1.228	5
*Sylvia communis*	6	40.8 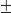 17.8	1.143	6
*Sylvia cantillans*	10	29.9 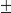 12.9	1.126	7
*Muscicapa striata*	4	48.0 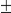 16.5	0.715	8
*Phoenicurus phoenicurus*	17	40.0 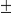 7.9	0.234	9
*Saxicola torquata*	3	52.0 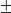 8.5	0.161	10
*Turdus philomelos*	4	60.5 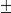 7.7	0.097	11
*Ficedula hypoleuca*	3	58.3 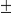 6.0	0.064	12
*Sylvia undata*	2	41.5 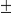 0.7	0.001	13

Gut passage times are expressed as mean (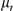) 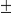 SD (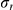) (in minutes). Kurtoses are predicted values based on assumed two-dimensional random movement (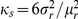).

As an example, we note large differences between two *Turdus* species (ranked 4 and 11) as potential long-distance dispersers although their mean retention times are nearly the same (see Table 3 and Figure S1). We add an important note of caution; we have ignored details such as relative abundance of disperser species, frequency of visits to the plant species and quantity of seeds consumed all of which will influence LDD. Our main purpose here is to illustrate predictive power of our simple model and it is possible to extend this formalism to normalize the effects of such detailed mechanisms for a fairer comparison.

Next, we ask whether the spatial range over which power-law dispersal may occur is significant in real systems. This may be obtained, under the assumptions of diffusive movement and gamma distributed retention times, by computing the cut-off distance (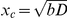). For birds of Table 2 and 3, we determine the range of the parameter 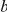 to be in 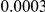 and 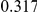 *day*. We predict that a cut-off distance of 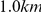 (or more), which is often considered a very large dispersal distance [11], can be achieved when diffusivity of birds is larger than 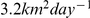. Since we lack the data for daily foraging movement of birds, we consider natal spreading rates of birds which are more commonly computed [35]; for example, natal diffusivity of obligate frugivores such as white-crowned pigeons in deciduous forests of Florida which has been estimated to be around 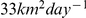 [45]. We emphasize that this is being used as a rough guide to estimate, but not as a substitute for, foraging patterns. Even if the diffusivity of daily foraging movement is smaller by an order of magnitude, it will be large enough to contribute to a substantial (*i.e.*, extending over 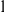 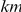 or more) power-law based seed dispersal. We, therefore, suggest that the spatial range over which animal mediated seed dispersal kernel exhibits power-law may indeed be realistically large for certain frugivorous species.

## Discussion

We present an analytical model that makes testable predictions relating animal movement behavior and seed retention time characteristics to seed dispersal patterns. We reveal that the scale, which is often employed as a measure of local dispersal, is determined by organisms' rate of movement and mean seed retention time. We then relate patterns of animal movement and gut retention times to different measures of LDD. First, we show that kurtosis or shape of the kernel can be driven by retention time variability of the dispersal units (seeds, pathogens, micro-organisms). Second, we determine that retention time variability can lead to a power-law dispersal with an exponential decay, thus having a tail that decays much slower than a Gaussian kernel. We also compute an absolute measure of LDD, defined as the number of seeds falling beyond a threshold distance, and show that larger the threshold distance, the larger the retention time variance at which LDD is maximized. We demonstrate the potential utility of our results in predicting key drivers of LDD by analyzing real data of frugivores from a Mediterranean forest.

Regardless of the specific mechanism of animal mediated dispersal, we expect that animals that cover larger distances and/or possesses larger seed retention times to more likely to facilitate the long-range transportation of dispersal units. However, it is not obvious how such factors translate into quantitative measures of a seed dispersal curve, such as its mean, scale, and kurtosis (or shape). Our analytical results on how animal behaviors such as rate of movement, and mean seed retention time influence the mean and scale of dispersal kernels are consistent with well established results in the literature on seed dispersal [8], [11], [28]. However, to the best of our knowledge, the variability in the seed retention time has not been identified in previous theoretical and/or empirical studies as an important driver of LDD. Even when such variation has been measured, the focus typically has been on movement patterns and/or large mean seed retention times [26], [28], [29]. We emphasize that it is not our claim that seed retention time variability is the only driver of LDD; instead, we argue that it is sufficient by itself to produce LDD.

We establish the generality and robustness of our results by showing that their qualitative features are largely independent of the details associated with different movement (diffusive, drift with diffusion, correlated random walk and home ranges) and seed transportation mechanisms (endozoochory and epizoochory); *i.e.*, although quantitative differences will occur, these do not affect the main conclusions of our paper. We note that, mathematically, movement and retention times both play an equivalent role in produce dispersal patterns (see Eq(1)). Therefore, we expect that variations in movement, as occurs when populations exhibit multiple modes/scales of movement characteristics ([14]; also see Text S7), will also drive LDD; this is consistent with other works which show that heterogeneous populations may exhibit leptokurtic and fat-tailed dispersal [14], [15], [33]. In addition to our analysis of seed retention times in frugivorous birds of Mediterranean forests, we draw attention to a recent study on an Amazonean frugivore that exhibits huge variations in both movement patterns and gut retention times, and can disperse seeds to extremely large distances [32]. Variability in individual retention times is an inescapable feature of the natural world and together with variations occurring from heterogeneity in movement and population structures, the chances of animal mediated LDD will only enhance.

In this work our aim was to identify minimal features of two key animal behaviors that can explain the large scale phenomenon of LDD. Details such as quantity of seed consumed, relative density of different vectors, habitat quality as well as post dispersal processes such as differential survival rates, germination, etc will all influence the spread and spatial structure of populations in important ways [8], [24]–[31], [46]. Future work can focus on an elaborate testing of our predictions, extend our analytical model to include more complex individual behaviors, heterogeneity in population structure and landscape characteristics as well as how these may affect eventual population and community dynamics.

In summary, we presented a simple analytical study providing clear and empirically testable links between animal movement, seed retention times, and the long-distance dispersal of seeds. A novel prediction of our study is that naturally occurring variations in the retention times of dispersal units by dispersal vectors can lead to long-distance dispersal, as measured through kurtosis, power-law dispersal and/or absolute number of long dispersal events. Such variations may arise, depending on the system and scales studied, at the individual or the population level, or at the community level (*i.e.*, across different species of dispersers). Using empirical data sets we illustrated the importance of variability in seed retention time for predicting the vectors that may potentially drive LDD of seeds. The model framework is general enough to be applicable to other important areas of vector mediated dispersal in ecology such as the spread of diseases. Being able to identify dispersal agents having highly variable retention times of their dispersal units may aid in the design of conservation strategies or the prevention of disease spread.

## Supporting Information

Text S1 Summary statistics of the seed dispersal kernel.
(PDF)

Text S2 Diffusive movement in one dimension.
(PDF)

Text S3 Diffusive movement in two dimensions.
(PDF)

Text S4 Diffusive movement in two dimensions with drift.
(PDF)

Text S5 Correlated random walks in one dimension.
(PDF)

Text S6 Random movement in a home-range.
(PDF)

Text S7 Power-law movement.
(PDF)

Text S8 Seed retention time distribution.
(PDF)

Text S9 Absolute measure of long-distance dispersal.
(PDF)

Figure S1 Predicted kurtosis from empirical data sets.
(PDF)
