# Supplementary material for: Retention Time Variability as a Mechanism for Animal Mediated Long-Distance Dispersal
Source: PLoS One. 2011 Dec 14;6(12):e28447. doi: 10.1371/journal.pone.0028447 (PMC3237446; doi:10.1371/journal.pone.0028447)
Supplement: Text S1 — Summary statistics of the seed dispersal kernel. (PDF) [file pone.0028447.s001.pdf]

Text S1: Summary statistics of the seed dispersal kernel

Here, we present an analytical method to connect the moments of a seed dispersal kernel (P_s) with the time-dependent moments of an animal movement pattern (P_m) and the distribution of seed retention time (P_r).

Suppose that $\Omega \in \mathbf{R}^d$, where $d \in \{1, 2\}$ is the number of spatial dimensions. In general, the mean, variance, and "excess" kurtosis of $P_s(\mathbf{x})$ in the x_i-direction ($i = 1, 2$) are defined to be

$$\begin{aligned} \mu_{si} &= \int_\Omega x_i P_s(\mathbf{x})\, d\mathbf{x} \\ \sigma_{si}^2 &= \int_\Omega (x_i - \mu_{si})^2 P_s(\mathbf{x})\, d\mathbf{x} \\ \kappa_{si} &= \frac{1}{(\sigma_{si})^4} \int_\Omega (x_i - \mu_{si})^4 P_s(\mathbf{x})\, d\mathbf{x} - 3 \end{aligned} \tag{1}$$

The constant 3 that appears in the equation for κ_{si} is present so that if P_s is Gaussian in the x_i-direction then $\kappa_{si} = 0$ or, in other words, it is a measurement relative to that of a Gaussian kernel. When $d = 2$, the covariance of P_s in the two directions x_1 and x_2 is defined to be

$$\sigma_{s12} = \int_\Omega (x_1 - \mu_{s1})(x_2 - \mu_{s2}) P_s(\mathbf{x})\, d\mathbf{x} \tag{2}$$

By expanding the polynomials that appear in the different integrands, all summary statistics can be expressed as summation of the moments $\int_\Omega x_i^m x_j^n P_s(\mathbf{x})\, d\mathbf{x}$ of P_s with appropriate coefficients. For example, if we expand the integrand of Eq (2) we obtain

$$\sigma_{s12} = \int_\Omega (x_1 x_2 - \mu_{s1} x_2 - \mu_{s2} x_1 + \mu_{s1}\mu_{s2}) P_s(\mathbf{x})\, d\mathbf{x} \tag{3}$$

where the first term on the right hand side is a moment term with ($m = 1, n = 1$), the second with ($m = 0, n = 1$), the third with ($m = 1, n = 0$) and the last term with ($m = 0, n = 0$). Likewise, other summary statistics can also be written as combinations of different moment terms.

In general, the (m, n)th moment of P_s can be found by substituting Eq (1*) (the asterisk symbol $*$ denotes main text) into the preceding moment formula and then changing the order of integration,

$$\int_\Omega x_i^m x_j^n P_s(\mathbf{x})\, d\mathbf{x} = \int_0^\infty \left(\int_\Omega x_i^m x_j^n P_m(\mathbf{x}, t)\, d\mathbf{x} \right) P_r(t)\, dt$$

Denoting the (m, n)th moment of a distribution $P_\bullet$ by $\mu_{\bullet ij}^{mn}$, we obtain the following important relation between the moments of P_s and P_m,

$$\mu_{sij}^{mn} = \int_0^\infty \mu_{mij}^{mn}(t) P_r(t)\, dt \tag{4}$$

Thus, the moments of P_s can always be computed provided that P_r and the (time-dependent) moments of P_m are known. It is not necessary to know P_m in full.

For notational simplicity we will write $\mu_{\bullet ij}^{mn}$ as $\mu_\bullet^m$ when $d = 1$, and we will write it as $\mu_{\bullet i}^m$ or $\mu_{\bullet j}^n$ when $d = 2$ and $mn = 0$. In the latter case, we will omit the remaining superscript if it is equal to 1.
