# Supplementary material for: Retention Time Variability as a Mechanism for Animal Mediated Long-Distance Dispersal
Source: PLoS One. 2011 Dec 14;6(12):e28447. doi: 10.1371/journal.pone.0028447 (PMC3237446; doi:10.1371/journal.pone.0028447)
Supplement: Text S2 — Diffusive movement in one dimension. (PDF) [file pone.0028447.s002.pdf]

Text S2: Diffusive movement in one dimension

Suppose first that animals perform random walks in $\Omega = \mathbf{R}$ (*i.e.,* one dimension) and let $P_m(x,t)$ be the probability density of an animal being at location $x \in \Omega$ at time $t \geq 0$. The initial value problem that determines the evolution of P_m is given by a diffusion equation

$$\frac{\partial P_m}{\partial t} = D\frac{\partial^2 P_m}{\partial x^2}, \quad P_m(x,0) = \delta(x)$$

and its solution is

$$P_m(x,t) = \frac{1}{\sqrt{4\pi D t}} \exp\Big(-\frac{x^2}{4Dt}\Big), \quad x \in \Omega \text{ and } t > 0 \tag{1}$$

The graph of this function is a Gaussian curve that expands because of diffusion.

Mean of P_s: It follows from Eq (4) of Text S1 and the fact that P_m is an even function of x that

$$\mu_s = \int_0^\infty \mu_m(t) P_r(t)\, dt = \int_0^\infty \Big(\int_{-\infty}^\infty x P_m\, dx\Big) P_r(t)\, dt = \int_0^\infty 0 \cdot P_r(t)\, dt = 0 \tag{2}$$

Scale of P_s: Substituting Eqs (1), (2), and (1*) into Eq (1) of Text S1 yields

$$\sigma_s^2 = \int_{-\infty}^\infty (x-\mu_s)^2 P_s\, dx = \int_0^\infty \Big(\int_{-\infty}^\infty x^2 P_m\, dx\Big) P_r(t)\, dt = \int_0^\infty (2Dt) P_r(t)\, dt = 2D\mu_r \tag{3}$$

Shape of P_s: Upon substituting Eqs (1), (2), (3) and Eq (1*) into Eq (1) of Text S1 we obtain

$$\begin{aligned}
\kappa_s &= \frac{1}{\sigma_s^4}\int_{-\infty}^\infty (x-\mu_s)^4 P_s ds - 3 = \frac{1}{\sigma_s^4}\int_0^\infty \Big(\int_{-\infty}^\infty x^4 P_m dx\Big) P_r(t) dt - 3 && (4)\\
&= \frac{1}{\sigma_s^4}\int_0^\infty (12D^2t^2) P_r(t)\, dt - 3 = \frac{12D^2}{(2D\mu_r)^2}(\mu_r^2 + \sigma_r^2) - 3 && (5)\\
&= \frac{3\sigma_r^2}{\mu_r^2} && (6)
\end{aligned}$$

Note that in our derivation of expressions for the summary statistics (*i.e.,* mean, scale and kurtosis above), we did not assume any specific distribution for retention time (P_r).

Furthermore, a counterintuitive feature of our results is that the kurtosis (a key measure of LDD) does not depend on the spatial spreading rate of the animal species (*i.e.,* the diffusion constant D).
