# Supplementary material for: Retention Time Variability as a Mechanism for Animal Mediated Long-Distance Dispersal
Source: PLoS One. 2011 Dec 14;6(12):e28447. doi: 10.1371/journal.pone.0028447 (PMC3237446; doi:10.1371/journal.pone.0028447)
Supplement: Text S3 — Diffusive movement in two dimensions. (PDF) [file pone.0028447.s003.pdf]

Text S3: Diffusive movement in two dimensions

Suppose animals perform random walks in $\Omega = \mathbf{R}^2$ (*i.e.*, two dimensions) and let $P_m(x_1, x_2, t)$ be the probability density of an animal being at location $\mathbf{x} = (x_1, x_2) \in \Omega$ at time $t \geq 0$. The initial value problem that determines the evolution of P_m is now [1]

$$\frac{\partial}{\partial t} P_m = D\Big(\frac{\partial^2}{\partial x_1^2} + \frac{\partial^2}{\partial x_2^2}\Big) P_m, \quad P_m(x_1, x_2, 0) = \delta(x_1)\delta(x_2) \tag{1}$$

and its solution is

$$P_m(x_1, x_2, t) = \frac{1}{4\pi D t} \exp\Big(- \frac{x_1^2 + x_2^2}{4Dt}\Big), \quad (x_1, x_2) \in \Omega \text{ and } t > 0 \tag{2}$$

The graph of this function is a 2-D Gaussian that expands because of diffusion.

Mean of P_s: It follows from Eq (4) of Text S1 and the fact that P_m is an even function of x_i that

$$\mu_{si} = \int_0^\infty \mu_{mi}(t) P_r(t)\, dt = \int_0^\infty \Big(\int_{-\infty}^\infty \Big(\int_{-\infty}^\infty x_i P_m \, dx_i \Big) dx_j \Big) P_r(t)\, dt = 0 \tag{3}$$

Scale of P_s: Substituting Eqs (2), (3), and (1*) into Eq (**??**) we obtain

$$\sigma_{si}^2 = \int_0^\infty \Big(\int_{-\infty}^\infty \int_{-\infty}^\infty x_i^2 P_m \, dx_1 \, dx_2 \Big) P_r(t)\, dt = \int_0^\infty (2Dt) P_r(t)\, dt = 2D\mu_r \tag{4}$$

We define the *total scale* of P_s to be

$$\sigma_s^2 = \sum_{i=1}^{2} \sigma_{si}^2 = 4D\mu_r$$

Shape and total kurtosis of P_s: Substituting Eqs (2), (3), (4), and (1*) into Eq (1) of Text S1 produces (see Text S2 for similar algebraic steps)

$$\kappa_{si} = \frac{1}{\sigma_{si}^4} \int_{-\infty}^\infty \int_{-\infty}^\infty x_i^4 P_s \, dx_1 \, dx_2 - 3 = \frac{3\sigma_r^2}{\mu_r^2}$$

We define the *total kurtosis* of P_s to be

$$\kappa_s = \sum_{i=1}^{2} \kappa_{si} = \frac{6\sigma_r^2}{\mu_r^2}$$

Covariance of P_s: As P_m is constant for fixed $x_1^2 + x_2^2$, the covariance of P_s is

$$\sigma_{s12} = \int_0^\infty \Big(\int_{-\infty}^\infty \int_{-\infty}^\infty x_i x_j P_m \, dx_1 \, dx_2 \Big) P_r(t)\, dt = \int_{-\infty}^\infty 0 \cdot P_r(t)\, dt = 0$$

Note that, until here, we have not made any assumptions on the full form of P_r. Therefore, as in for the one dimensional case, the above results are generally applicable to any retention time pattern for a diffusively moving organism in two dimensions.

Form of P_s To find an expression for P_s, we substitute Eqs (2) and (3*) into Eq (1*) to obtain [2],

$$P_s(x_1, x_2) = \frac{A_0}{x_c^{a+1}} x^{a-1} K_{a-1}\left(\frac{x}{x_c}\right), \quad (x_1, x_2) \in \Omega$$

Here, A_0 is a positive constant (depending only on a), $x_c = \sqrt{bD}$, and $x = \sqrt{x_1^2 + x_2^2}$. At large distances, we have the approximation

$$P_s(x) \approx \frac{B_0}{x_c^{a+\frac{1}{2}}} x^{a-\frac{3}{2}} e^{-\frac{x}{x_c}}, \quad x \gg x_c$$

As in one dimensional case of Eqs (6*) and (7*), P_s exhibits power-law with an exponential cut-off.

References

[1] Okubo A, Levin SA (2001) *Diffusion and ecological problems: modern perspectives.* New York: Springer-Verlag.

[2] Wolfram Research Inc (2004) Mathematica, Version 5.2. Champaign, IL: Wolfram Research, Inc.
