# Supplementary material for: Retention Time Variability as a Mechanism for Animal Mediated Long-Distance Dispersal
Source: PLoS One. 2011 Dec 14;6(12):e28447. doi: 10.1371/journal.pone.0028447 (PMC3237446; doi:10.1371/journal.pone.0028447)
Supplement: Text S4 — Diffusive movement in two dimensions with drift. (PDF) [file pone.0028447.s004.pdf]

Text S4: Diffusive movement in two dimensions with drift

Suppose animals perform random walks with drift in $\Omega = \mathbf{R}^2$ and let $P_m(x_1, x_2, t)$ be the probability density of an animal being at location $\mathbf{x} = (x_1, x_2) \in \Omega$ at time $t \geq 0$. The initial value problem that determines the evolution of P_m is now an advection-diffusion equation [1]

$$\frac{\partial}{\partial t} P_m = D\Big(\frac{\partial^2}{\partial x_1^2} + \frac{\partial^2}{\partial x_2^2}\Big) P_m - \Big(v_1 \frac{\partial}{\partial x_1} + v_2 \frac{\partial}{\partial x_2}\Big) P_m, \quad P_m(x_1, x_2, 0) = \delta(x_1)\delta(x_2)$$

Here, v_i is the net velocity of animals in the x_i-direction. A drift can result for a variety of reasons, including the presence of wind or water, an animal's migratory behavior, or the influence of an elevational gradient. Depending on the relative magnitude of the diffusion rate D and the advection/drift terms (v_1 and v_2), the movement can be dominated by random motion, directed motion, or both. The solution of the equation above is

$$P_m(x_1, x_2, t) = \frac{1}{4\pi D t} \exp\Big(- \frac{(x_1 - v_1 t)^2 + (x_2 - v_2 t)^2}{4Dt} \Big), \quad (x_1, x_2) \in \Omega \text{ and } t > 0 \tag{1}$$

The graph of this function is a 2-D Gaussian that expands because of diffusion and whose center moves in the direction (v_1, v_2) with speed $v = \sqrt{v_1^2 + v_2^2}$.

Mean, scale, shape, and covariance of P_s: It can be shown using arguments similar to those used before that

$$\mu_{si} = v_i \mu_r, \quad \sigma_{si}^2 = 2D\mu_r + v_i^2 \sigma_r^2 \quad \text{and} \quad \kappa_{si} = \frac{6\sigma_r^2}{\mu_r^2} \left\{ 1 - 2\left(2 + \frac{v_i^2 \sigma_r^2}{D\mu_r}\right)^{-2} \right\}$$

Furthermore, the covariance of P_s is $v_1 v_2 \sigma_r^2$. Here, as in one and two dimensional cases, we have not yet made any assumptions on the full form of P_r. Therefore, the above results on moments of seed dispersal kernel are generally applicable to organisms moving via diffusion with drift with any retention time pattern (P_r).

Form of P_s: To find an expression for P_s, we substitute Eqs (1) and (3*) into Eq (1*) to obtain [2],

$$P_s(x_1, x_2) = \frac{A_0}{x_c} \left(\frac{x_c}{bD}\right)^a x^{a-1} \exp\left(\frac{x_1 v_1 + x_2 v_2}{2D}\right) K_{a-1}\left(\frac{x}{x_c}\right)$$

Here, A_0 is a positive constant (depending only on a), $x_c = \left(\frac{4bD^2}{4D+bv^2}\right)^{\frac{1}{2}}$, and $x = \sqrt{x_1^2 + x_2^2}$. In a polar direction θ (where the angle is taken with respect to the direction of the drift), we approximate P_s at large distances by

$$P_s(x, \theta) \approx \frac{B_0}{x_c} \left(\frac{x_c}{bD}\right)^a x^{a - \frac{3}{2}} \exp\left(-x\left\{\frac{1}{x_c} - \frac{v\cos\theta}{2D}\right\}\right), \quad x \gg x_c$$

Therefore, we conclude that P_s exhibits power-law with an exponential cut-off. It is easy to see that the cut-off distance x_c can be no larger than $\max\{\sqrt{bD}, \frac{2D}{v}\}$, and that it approaches one of these values as the corresponding mode of transport becomes dominant. That is, if random motion dominates ($bv^2 \ll 4D$) then $x_c \approx \sqrt{bD}$ and if directed motion dominates ($bv^2 \gg 4D$) then $x_c \approx \frac{2D}{v}$. See Fig S(1) for features of P_s for different values of variations in seed retention times (σ_r^2), which is qualitatively similar previous random walk models without drift (Figure 3*(a-b)).

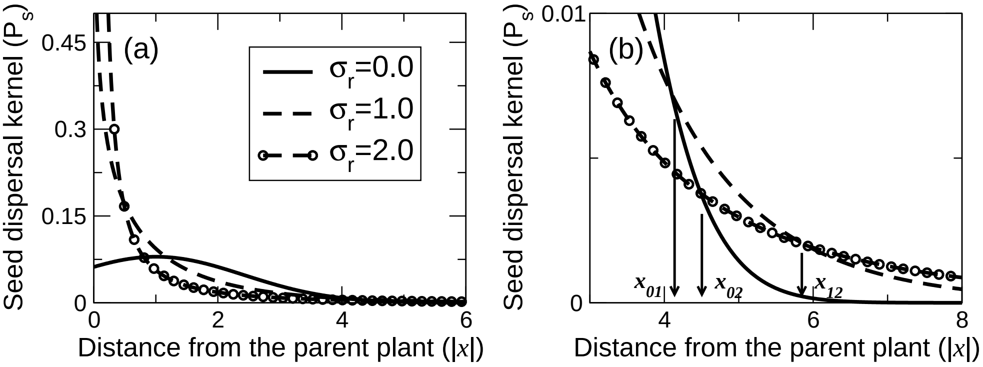

Figure S 1: Diffusion with drift in two dimensions. (a) The seed dispersal kernel as a function of distance from the source tree ($|x|$) and standard deviation in seed retention time (σ_r). The case $\sigma_r = 0$ corresponds to a Gaussian kernel. (b) The seed dispersal kernel at larger distances. The symbol x_{ij} (*e.g.,* x_{01}) indicates the distance at which a seed dispersal kernel with $\sigma_r = j$ (*e.g.,* $\sigma_r = 1$) begins to have more long distance dispersal events than a seed dispersal kernel with $\sigma_r = i$ (*e.g.,* $\sigma_r = 0$). Note that $x_{01} < x_{02} < x_{12}$ ($x_{01} \approx 4.1, x_{02} \approx 4.3, x_{12} \approx 5.9$). Parameters: $D = 1.0$, $\mu_r = 1.0$, $v_1 = 1$, and $v_2 = 0$.

References

[1] Okubo A (1986) Dynamical aspects of animal grouping: swarms, schools, flocks, and herds. Adv Biophys 22: 1.

[2] Wolfram Research Inc (2004) Mathematica, Version 5.2. Champaign, IL: Wolfram Research, Inc.
