# Supplementary material for: Retention Time Variability as a Mechanism for Animal Mediated Long-Distance Dispersal
Source: PLoS One. 2011 Dec 14;6(12):e28447. doi: 10.1371/journal.pone.0028447 (PMC3237446; doi:10.1371/journal.pone.0028447)
Supplement: Text S5 — Correlated random walks in one dimension. (PDF) [file pone.0028447.s005.pdf]

Text S5: Correlated random walks in one dimension

Suppose animals perform correlated random walks in one dimension (*i.e.,* $\Omega = \mathbf{R}$) and let $P_m(x,t)$ be the probability density of an animal being at location $x \in \Omega$ at time $t \geq 0$ (we restrict our attention to one dimension for analytical tractability). The initial value problem that determines the evolution of P_m is given by a telegraph equation [1]:

$$\frac{\partial^2 P_m}{\partial t^2} + \frac{2}{\tau}\frac{\partial P_m}{\partial t} = u^2 \frac{\partial^2 P_m}{\partial x^2}, \qquad P_m(x,0) = \delta(x) \quad \text{and} \quad \frac{\partial P_m}{\partial t}(x,0) = 0 \tag{1}$$

Here, τ is the characteristic time an animal moves before changing directions and u is its speed. As correlation time (τ) approaches zero, Eq (1) reduces to a diffusion equation in which D equals the limit of $\frac{1}{2}u^2\tau$ as $\tau \to 0$ and $u \to \infty$. The solution of (1) is [2]:

$$P_m(x,t) = \begin{cases} e^{-\frac{t}{\tau}}\{\delta(x-ut) + \delta(x+ut) + \frac{1}{2u\tau}[I_0(z) + \frac{t}{\tau z}I_1(z)]\}, & |x| < ut \\ 0, & |x| > ut \end{cases}$$

where $z = \frac{1}{\tau}\sqrt{t^2 - \frac{x^2}{v^2}}$ and I_0 and I_1 are the modified Bessel functions of the first kind (there is no known closed form solution to the telegraph equation in two dimensions). Below, we compute the summary statistics of P_s.

Mean of P_s: Multiply Eq (1) by x and integrate over Ω to get

$$\mu_m''(t) + \frac{2}{\tau}\mu_m'(t) = 0, \quad \mu_m(0) = \mu_m'(0) = 0$$

Here, we have used the fact that P_m and $\partial P_m/\partial x$ both approach zero as $x \to \pm\infty$. It follows from the uniqueness of solutions to differential equations that $\mu_m = 0$, and hence from Eq (1) of Text S1 that $\mu_s = 0$.

Scale of P_s: To determine σ_s^2 we first determine $\mu_m^2(t)$ (the second moment of P_m, not the square of its first moment). Repeating the same procedure as above but multiplying Eq (1) by x^2 instead of x yields

$$(\mu_m^2)''(t) + \frac{2}{\tau}(\mu_m^2)'(t) = 2u^2, \quad \mu_m^{12}(0) = (\mu_m^2)'(0) = 0$$

whose solution is

$$\mu_m^2(t) = \frac{u^2\tau^2}{2}\Big(-1 + \frac{2t}{\tau} + e^{-\frac{2t}{\tau}}\Big) \tag{2}$$

Straightforward calculations involving Eqs (1) and (4) of Text S1, (2), and (3*) lead to

$$\sigma_s^2 = \mu_s^2 = \int_0^\infty \mu_m^2(t)P_r(t)\,dt = \frac{u^2\tau^2}{2}\left(-1 + \frac{2ab}{\tau} + \left(1 + \frac{2b}{\tau}\right)^{-a}\right)$$

where a and b are parameters of gamma distributed retention times (P_r) of Eq (3*). In comparing this result with the corresponding one for random motion, and to identify the effects of correlation, it will be convenient to introduce the dimensionless quantities $\omega = \frac{\mu_r}{\tau} = \frac{ab}{\tau}$ and $\xi^2 = \frac{\sigma_r^2}{\mu_r^2} = \frac{1}{a}$. In so doing, we fix D to be a constant and choose u and τ such that $\frac{1}{2}u^2\tau = D$. With these substitutions,

$$\sigma_s^2(D\tau, \omega, \xi^2) = D\tau\left(-1 + 2\omega + (1 + 2\omega\xi^2)^{-1/\xi^2}\right) \tag{3}$$

Shape of P_s: To determine κ_s we first determine $\mu_m^4(t)$ (the fourth moment of P_m). Multiplying Eq (1) by x^4 and then integrating over Ω produces

$$(\mu_m^4)''(t) + \frac{2}{\tau}(\mu_m^4)'(t) = 12u^2\mu_m^2, \quad \mu_m^4(0) = (\mu_m^4)'(0) = 0$$

The solution of this differential equation is

$$\mu_m^4(t) = \frac{3u^4\tau^4}{2}\left(3 + \frac{2t}{\tau}\left(\frac{t}{\tau} - 2\right) - \left(3 + \frac{2t}{\tau}\right)e^{-\frac{2t}{\tau}}\right) \tag{4}$$

Straightforward calculations involving Eq (4) of Text S1, (4), and (3*) lead to

$$\mu_s^4 = \frac{3u^4\tau^4}{2}\left(3 - \frac{4ab}{\tau} + \frac{2a(a+1)b^2}{\tau^2} - \left(3 + \frac{2(a+3)b}{\tau}\right)\left(1 + \frac{2b}{\tau}\right)^{-1-a}\right)$$

Utilizing the dimensionless parameters,

$$\mu_s^4 = 6(D\tau)^2\left(3 - 4\omega + 2\omega^2(1+\xi^2) - \left(3 + 2\omega(1+3\xi^2)\right)\left(1+2\omega\xi^2\right)^{-1-\frac{1}{\xi^2}}\right) \tag{5}$$

Eq (1) of Text S1, (3), and (5) and the relation $\mu_s = 0$ together imply that

$$\kappa_s(\omega, \xi^2) = \frac{\mu_s^4}{\sigma_s^4} - 3 = 6\left\{\frac{3 - 4\omega + 2\omega^2(1+\xi^2) - \left(3 + 2\omega(1+3\xi^2)\right)\left(1+2\omega\xi^2\right)^{-1-\frac{1}{\xi^2}}}{\left(-1 + 2\omega + (1+2\omega\xi^2)^{-1/\xi^2}\right)^2}\right\} - 3$$

Although the shape (κ_s) of P_s does not depend directly on $D = \frac{1}{2}u^2\tau$ (as was also the case with random motion), it does depend on correlation time τ via ω. See the panel in Fig S(1)). Also note that, unlike in previous random walk models where summary statistics of P_s were general with respect to P_r, the expressions for the scale and shape above depend on explicit form of P_r to be a gamma distribution.

Form of P_s: Although we are unable to obtain a closed form for the seed dispersal kernel (P_s), we can find it using numerical integration. See Fig S(2).

References

[1] Othmer HG, Dunbar SR, Alt W (1988) Models of dispersal in biological systems. J Math Biol 26: 263–298.

[2] Morse P, Feshbach H, Hill E (1954) In: Methods of theoretical physics, volume 22. p. 410.

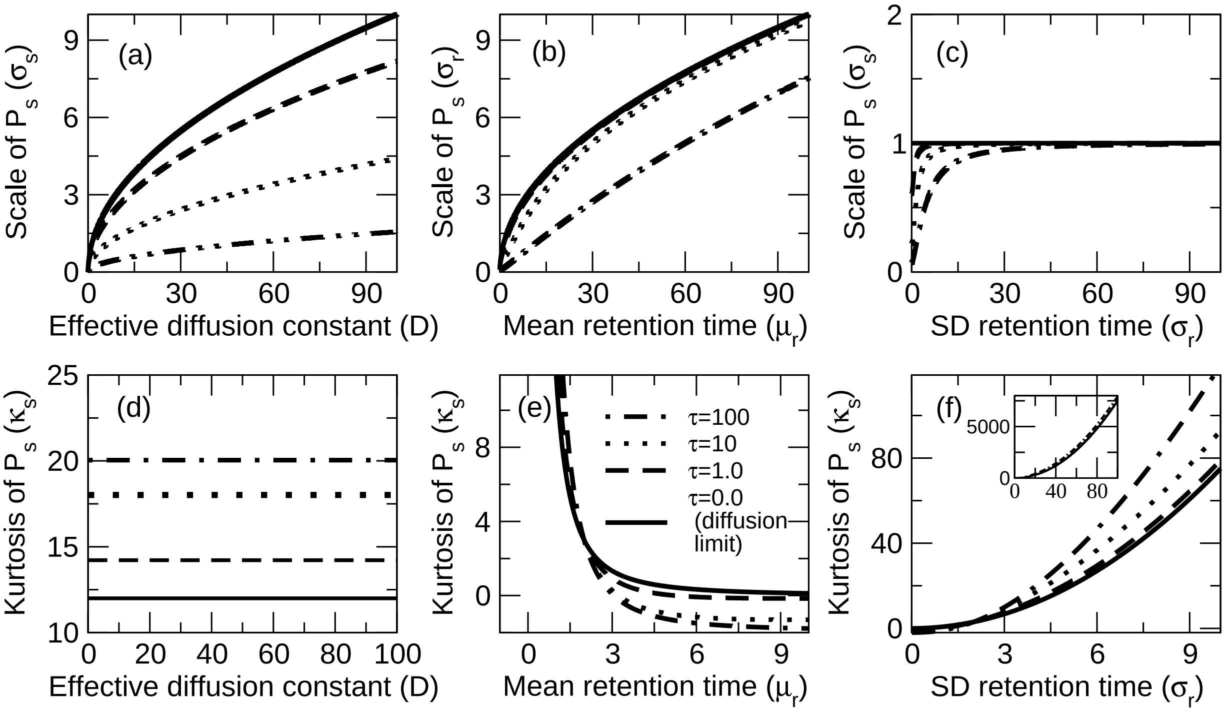

Figure S1: Scale (σ_s) and kurtosis (κ_s) of the seed dispersal kernel for animals that move according to correlated random walks (CRW). Scale as a function of: (a) The effective diffusion constant, $D = \frac{1}{2}u^2\tau$; Parameters: $\mu_r = 0.5$ and $\sigma_r = 1.0$. (b) Mean seed retention time, μ_r; Parameters: $D = 0.5$ and $\sigma_r = 1.0$. (c) Standard deviation (SD) of seed retention time, σ_r; Parameters: $D = 1.0$ and $\mu_r = 1.0$. Excess kurtosis as a function of: (d) The effective diffusion constant, D; Parameters: $\mu_r = 1.0$ and $\sigma_r = 2.0$. (e) Mean seed retention time, μ_r; Parameters: $D = 1.0$ and $\sigma_r = 2.0$. (f) Standard deviation (SD) of seed retention time, σ_r; Parameters: $D = 1.0$ and $\mu_r = 2.0$. Inset in (f) shows that the trend of κ_s over large scales of σ_r is qualitatively unaffected by the choice of the correlation time scale (τ).

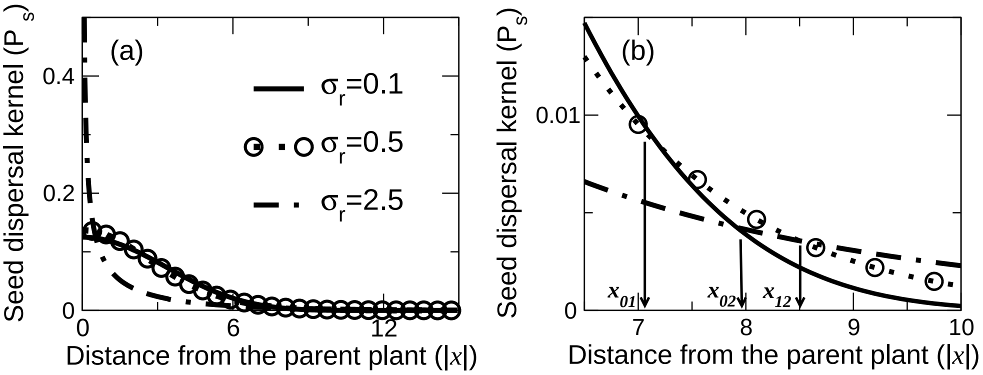

Figure S2: Correlated random walk in one dimension. (a) The seed dispersal kernel as a function of distance from the source tree ($|x|$) and standard deviation in seed retention time (σ_r). (b) The seed dispersal kernel at larger distances. The larger the σ_r, the more frequent the LDD events. Note that $x_{01} < x_{02} < x_{12}$ ($x_{01} \approx 7.1, x_{02} \approx 8.0, x_{12} \approx 8.5$). Parameters: $\tau = 1.0$ and $\mu_r = 10.0$.
