# Supplementary material for: Retention Time Variability as a Mechanism for Animal Mediated Long-Distance Dispersal
Source: PLoS One. 2011 Dec 14;6(12):e28447. doi: 10.1371/journal.pone.0028447 (PMC3237446; doi:10.1371/journal.pone.0028447)
Supplement: Text S6 — Random movement in a home-range. (PDF) [file pone.0028447.s006.pdf]

Text S6: Random movement in a home-range

Animals often have a home-range and/or a preferred territory for their movement [1; 2]. To model this we suppose animals performing random walks in one dimension ($\Omega = \mathbf{R}$) are consistently attracted to a fixed point in space, such as their resting site (as with CRW, we restrict our analysis to one dimension for analytical tractability). We assume that the resting site is located at the origin and, much like a ball in a gravity well, the farther the animal is from its resting site, the faster its rate of return. Such a motion with stochastic effects may be modeled by the Ornstein-Uhlenbeck process [3]

$$\frac{dx}{dt} = -\gamma x + \eta(t)$$

where x is position, γ^{-1} is a measure of average return-time to the resting site, and $\eta(t)$ is a Gaussian white noise with mean zero and variance $2D$.

Let $P_m(x,t)$ be the probability density of an animal being at location $x \in \Omega$ at time $t \geq 0$. The initial value problem that determines the evolution of P_m is given by

$$\frac{\partial P_m}{\partial t} = \frac{\partial(\gamma x P_m)}{\partial t} + D\frac{\partial^2 P_m}{\partial x^2} \qquad P_m(x,0) = \delta(x) \quad \text{and} \quad \frac{\partial P_m}{\partial t}(x,0) = 0$$

and its solution is [3]

$$P_m(x,t) = \frac{1}{\sqrt{2\pi\sigma(t)^2}} \exp\Big(-\frac{x^2}{2\sigma(t)^2}\Big) \qquad \text{where} \qquad \sigma(t) = \sqrt{\frac{D}{\gamma}(1 - e^{-2\gamma t})} \tag{1}$$

Below, we compute the summary statistics of P_s.

Mean of P_s: Multiply Eq (1) by x and integrate over Ω to get

$$\mu_m(0) = 0$$

It then follows from Eq (1) of Text S1 that $\mu_s = 0$.

Scale of P_s: To determine σ_s^2 we first note that the second moment of P_m is

$$\mu_m^2(t) = \sigma(t)^2 = \frac{D}{\gamma}(1 - e^{-2\gamma t}) \tag{2}$$

Straightforward calculations involving Eqs 1, 4 of Text S1, and (2), and (3*) lead to

$$\sigma_s^2(D,\gamma) = \frac{D}{\gamma}(1 - (1 + 2\gamma b)^{-a}) \tag{3}$$

where a and b are parameters of gamma distributed retention times of Eq (3*). By introducing the dimensionless quantities $z = \mu_r\gamma = ab\gamma$ and $\xi^2 = \frac{\sigma_r^2}{\mu_r^2} = \frac{1}{a}$, we obtain

$$\sigma_s^2(D/\gamma, z, \xi) = \frac{D}{\gamma}(1 - (1 + 2z\xi^2)^{-1/\xi^2})$$

Shape of P_s: To determine κ_s we first determine $\mu_m^4(t)$ (the fourth moment of P_m). Multiplying Eq (1) by x^4, integrating over Ω, and then solving for μ_s^4 produces

$$\mu_s^4 = \frac{2D^2}{\gamma^2}\Big(1 - 2(1+2\gamma b)^{-a} + (1+4\gamma b)^{-a})\Big)$$

Utilizing the dimensionless parameters z and ξ,

$$\mu_s^4 = \frac{3D^2}{\gamma^2}\Big(1 - 2(1+2z\xi^2)^{-1/\xi^2} + (1+4z\xi^2)^{-1/\xi^2})\Big) \tag{4}$$

Eq (1) of Text S1, (3), and (4) and the relation $\mu_s = 0$ together imply that

$$\kappa_s(z,\xi^2) = \frac{\mu_s^4}{\sigma_s^4} - 3 = 3\left\{\frac{1 - 2(1+2z\xi^2)^{-1/\xi^2} + (1+4z\xi^2)^{-1/\xi^2}}{(1-(1+2z\xi^2)^{-1/\xi^2})^2}\right\} - 3$$

As shown in Figures (2*) (recall that * represents the maintext) and (1) in this supplementary text, the scale and kurtosis converge to their counterparts in the diffusion model as $\gamma \to 0$.

Form of P_s: For this movement model we are unable to obtain a closed form for the seed dispersal kernel (P_s) but we can compute it using numerical integration (see Fig S(2)). We see that its qualitative features, that larger seed retention time variability leads to a larger dispersal probability beyond a certain distance, agrees with that of other movement models.

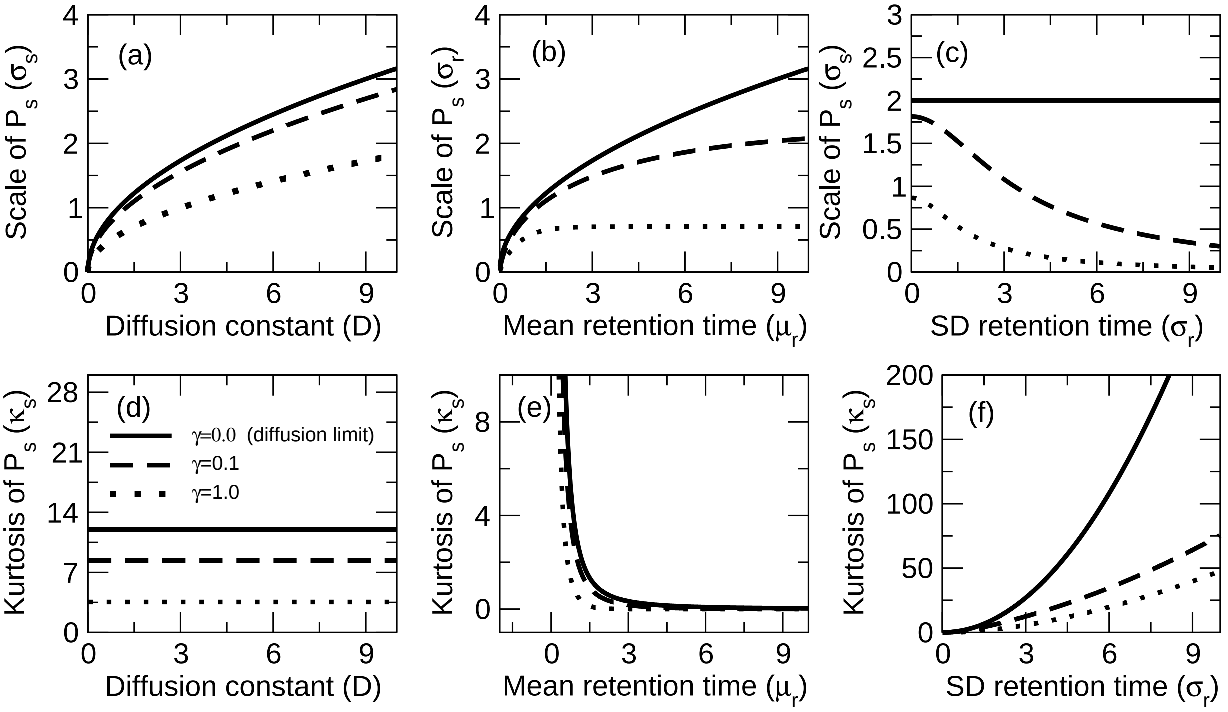

Figure S1: Scale (σ_s) and kurtosis (κ_s) of the seed dispersal kernel for animals moving in their home-rage. Scale as a function of: (a) The diffusion constant, D; Parameters: $\mu_r = 0.5$ and $\sigma_r = 1.0$. (b) Mean seed retention time, μ_r; Parameters: $D = 0.5$ and $\sigma_r = 1.0$. (c) Standard deviation (SD) of seed retention time, σ_r; Parameters: $D = 1.0$ and $\mu_r = 1.0$. Excess kurtosis as a function of: (d) The diffusion constant, D; Parameters: $\mu_r = 0.5$ and $\sigma_r = 1.0$. (e) Mean seed retention time, μ_r; Parameters: $D = 0.5$ and $\sigma_r = 1.0$. (f) Standard deviation (SD) of seed retention time, σ_r; Parameters: $D = 1.0$ and $\mu_r = 1.0$.

References

[1] Moorcroft PR, Lewis MA (2006) *Mechanistic home range analysis.* Princeton University Press.

[2] Borger L, Dalziel BD, Fryxell JM (2008) Are there general mechanisms of animal home range behaviour? A review and prospects for future research. Ecol Lett 11: 637–650.

[3] Gardiner CW (2003) *Handbook of stochastic methods for Physics, Chemistry and the Natural Sciences.* Springler-Verlag, 3rd edition.

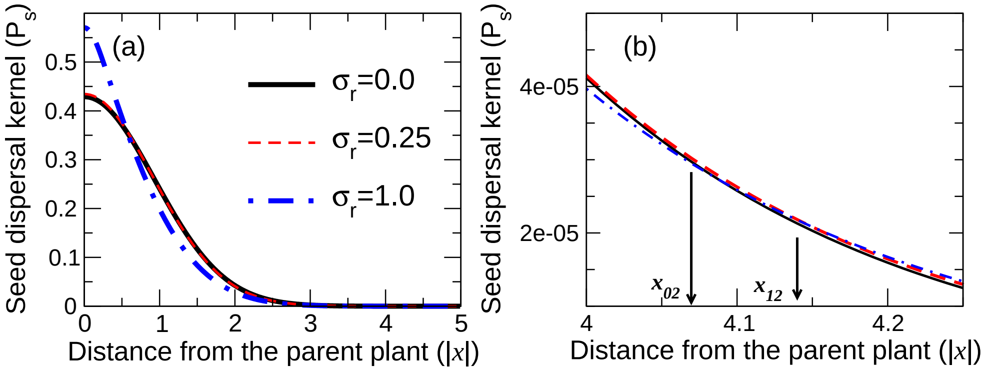

Figure S 2: Random walk in home-range. (a) The seed dispersal kernel as a function of distance from the source tree ($|x|$) and standard deviation in seed retention time (σ_r). The case $\sigma_r = 0$ corresponds to a Gaussian kernel. (b) The seed dispersal kernel at larger distances. The symbol x_{ij} (*e.g.*, x_{02}) indicates the distance at which a seed dispersal kernel with $\sigma_r = j$ (*e.g.*, $\sigma_r = 2$) begins to have more long distance dispersal events than a seed dispersal kernel with $\sigma_r = i$ (*e.g.*, $\sigma_r = 0$). Note that $x_{02} < x_{12}$. Parameters: $D = 1.0$, $\mu_r = 1.0$ and $\gamma = 1.0$.
