# Supplementary material for: Retention Time Variability as a Mechanism for Animal Mediated Long-Distance Dispersal
Source: PLoS One. 2011 Dec 14;6(12):e28447. doi: 10.1371/journal.pone.0028447 (PMC3237446; doi:10.1371/journal.pone.0028447)
Supplement: Text S7 — Power-law movement. (PDF) [file pone.0028447.s007.pdf]

Text S7: Power-law movement in two dimensions

Suppose now that animals move randomly in two dimensions but that individuals vary in their diffusion rate. In particular, we assume that their diffusivity D follows a Gamma distribution (with shape α and scale β). Then the tail of the movement pattern obeys [1]

$$P_m(x,t) \approx A_0 |x|^{\alpha - \frac{3}{2}} e^{-\frac{|x|}{\beta t}}, \quad |x| \gg 1$$

Here, A_0 is a constant that does not depend on x. As our interest is in determining whether a power-law displacement pattern can produce LDD of seeds in the absence of seed retention time variability ($\sigma_r^2 = 0$), we consider the special case in which $P_r(t) = \delta(t - T)$. Under this condition, Eq (1*) implies that

$$P_s(x) \approx A_0 |x|^{\alpha - \frac{3}{2}} e^{-\frac{|x|}{\beta T}}, \quad |x| \gg 1$$

which is indeed power-law seed dispersal kernel.

References

[1] Petrovskii S, Morozov A (2009) Dispersal in a Statistically Structured Population: Fat Tails Revisited. Am Nat 173: 278–289.
