# Supplementary material for: Retention Time Variability as a Mechanism for Animal Mediated Long-Distance Dispersal
Source: PLoS One. 2011 Dec 14;6(12):e28447. doi: 10.1371/journal.pone.0028447 (PMC3237446; doi:10.1371/journal.pone.0028447)
Supplement: Text S8 — Seed retention time distribution. (PDF) [file pone.0028447.s008.pdf]

Text S8: Seed retention time distribution (P_r)

We discuss the generality of results with respect to the choice of retention time distribution (P_r). For simplicity and analytical tractability, we assume that animals move diffusively in two dimensions. We begin by recalling that the mean, scale and shape (kurtosis) of the seed dispersal kernel (see Texts S2, S3 and S4) depend on the summary statistics of retention time distribution (P_r), but not on its specific form such as Gamma distribution. Therefore our results that LDD is driven by variation in seed retention time continues to hold.

We note that Gamma distribution has some specific features; a power-law decay with an exponential cut-off (see the term $t^{a-1}\exp(-t/b)$ in Eq (3*)) and it allows for arbitrarily large value of retention time. We ask to what extent our results that the seed dispersal kernel has a power-law distribution are dependent on these features of Gamma distribution.

To do so we briefly mention a recent study that we came across while writing our manuscript [1]. Authors in that study obtain expression for the spread of population density ($\tilde{n}(x,t)$) when individuals move diffusively in two dimensions and that the population is structured due to individual variations in diffusion constants (D),

$$\tilde{n}(x,t) = \int_0^\infty \frac{1}{4\pi Dt}\exp\Big(-\frac{x^2}{4Dt}\Big)\phi(D)dD \tag{1}$$

where $\phi(D)$ is the distribution of diffusivity constant in the population and $x = \sqrt{x_1^2 + x_2^2}$. For a range of distributions $\phi(D)$, they show that the spread (or dispersal) of organisms, at any given time t, exhibits an algebraic decay with distance (also see Text S7).

Note that they studied movement distribution of organisms but not the dispersal of units carried by moving organisms. By an interchange of variables D and t, and the corresponding distribution $\phi \to P_r$ in Eq (1), it is easy to see that we can map the movement distribution of organisms in their model to the seed dispersal kernel generated by diffusively moving animals in our model *i.e.,* $\tilde{n}(x,t) \to P_s(x,D)$, or explicitly,

$$P(x,t) = \int_0^\infty \frac{1}{4\pi tD}\exp\Big(-\frac{x^2}{4tD}\Big)P_r(t)dt$$

which is of the form Eq (1*) with $P_m(x,t)$ given by Eq (2*). Therefore we can borrow their arguments to test generality of our results with respect to P_r.

Gaussian for P_r: We consider a Gaussian distribution for P_r

$$P_r(t) = \frac{1}{\sqrt{2\pi\sigma_r^2}}\exp\Big(-\frac{(t-\mu_r)^2}{2\sigma_r^2}\Big) \tag{2}$$

which has a tail that decays at a rate faster than that of a Gamma distribution and contains no in-built power-law features. Here μ_r and σ_r are mean and SD of retention time, respectively. To find an expression for P_s we substitute Eq (2) of Text S3, and Eq (2) above in Eq (1*), *i.e.,*

$$P_s(x) = \int_0^\infty \frac{1}{4\pi Dt}\frac{1}{\sqrt{2\pi\sigma_r^2}}\exp\Big(-\frac{x^2}{4Dt}\Big)\exp\Big(-\frac{(t-\mu_r)^2}{2\sigma_r^2}\Big)$$

We can not obtain a closed form expression for the above integral. For large distances, however, it can be shown that [1]

$$P_s(x) = C_0 x^{-2/3} \exp\Big(-\frac{3}{4}\Big(\frac{x}{x_c}\Big)^{4/3}\Big)$$

where $x_c = \sqrt{2\sigma_r D}$ and C_0 is a constant depending on D and μ_r. This is indeed a power-law decay with a cut-off function ($\exp(-3/4(x/x_c)^{4/3})$) that is faster than an exponential but slower than a Gaussian.

Finite boundedness in retention time: It can be shown that if P_r is a distribution with finite bound on the retention time, it does not affect the qualitative nature of our results. We will still find a power-law P_s; but in comparison to results arising from Gamma distributed P_r, the range of spatial scales over which power-law occurs reduces. We refer readers to [1] for technical details of derivation of this result; note that they consider boundedness in diffusivity in their model.

References

[1] Petrovskii S, Morozov A (2009) Dispersal in a Statistically Structured Population: Fat Tails Revisited. Am Nat 173: 278–289.
