# Supplementary material for: Retention Time Variability as a Mechanism for Animal Mediated Long-Distance Dispersal
Source: PLoS One. 2011 Dec 14;6(12):e28447. doi: 10.1371/journal.pone.0028447 (PMC3237446; doi:10.1371/journal.pone.0028447)
Supplement: Text S9 — Absolute measure of long-distance dispersal. (PDF) [file pone.0028447.s009.pdf]

Text S9: Absolute measure of long distance dispersal

Based on [1], we define an absolute measure of long distance dispersal to be the proportion of seeds falling beyond a threshold distance. Mathematically we can write this as:

$$f_{ldd}(d_c, \sigma_r) = \int_{d_c}^{\infty} P_s(|x|; \sigma_r)\, dx \quad (1)$$

$$= P_s(|x| > d_c) = 1 - P_s(|x| \leq d_c) \quad (2)$$

where f_{ldd} is the absolute measure and d_c is the threshold dispersal distance. Although the seed dispersal kernel P_s, and consequently f_{ldd}, depends on many other animal behavior characteristics such as diffusion rate, mean seed retention time, etc, we focus only on the impact of variations (or SD) in the retention time and threshold dispersal distance (d_c).

Here, we describe the procedure we followed to obtain Fig 3*c (of the main text). We assume that animals move diffusively in a two dimensional environment. Next, we substitute Eq 2 of Text S3 for P_s into Eq (2) and use Mathematica to evaluate the integration numerically [2]. For a fixed value of d_c, we determine f_{ldd} as a function of σ_r (see Fig S1) and normalize it by the maximum value of f_{ldd}. We denote the normalized value of absolute LDD by $\hat{f}_{ldd}$. We repeat this procedure for many values of the threshold distance d_c.

For each value of the threshold dispersal distance d_c, we identify the value of σ_r^* that maximizes absolute LDD events, f_{ldd} (or where $\hat{f}_{ldd} = 1$). We join the values of σ_r^* to obtain a pitchfork like bifurcation pattern as a function of the threshold distance in Fig 3* (C) of the main text.

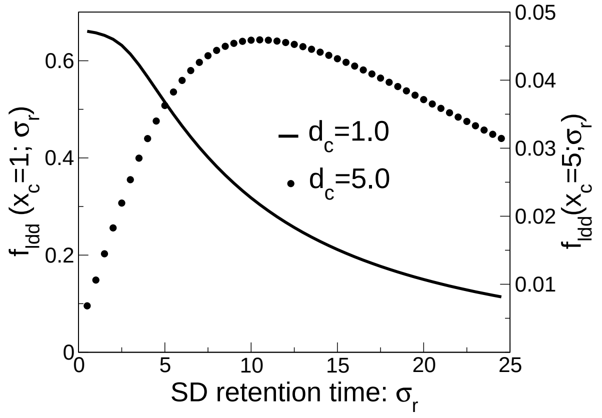

Figure S 1: Absolute measure of long distance dispersal. Parameters: $D = 1.0$ and $\mu_r = 1.0$.

References

[1] Nathan R, Schurr FM, Spiegel O, Steinitz O, Trakhtenbrot A, et al. (2008) Mechanisms of long-distance seed dispersal. Trends Ecol Evol 23: 638–647.

[2] Wolfram Research Inc (2004) Mathematica, Version 5.2. Champaign, IL: Wolfram Research, Inc.
