# Supplementary material for: Retention Time Variability as a Mechanism for Animal Mediated Long-Distance Dispersal
Source: PLoS One. 2011 Dec 14;6(12):e28447. doi: 10.1371/journal.pone.0028447 (PMC3237446; doi:10.1371/journal.pone.0028447)
Supplement: Figure S1 — Predicted kurtosis from empirical data sets. (PDF) [file pone.0028447.s010.pdf]

Figure S1: Predicted kurtosis from empirical data sets

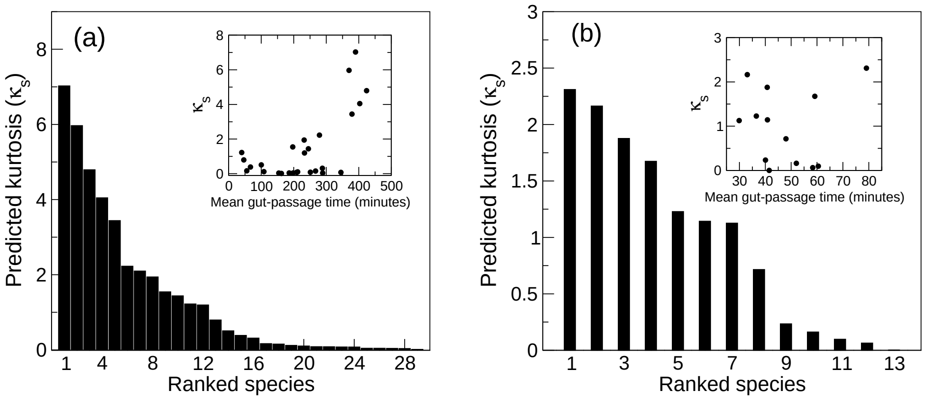

Figure S 1: Ranked predicted kurtosis ($\kappa_s = 6\,\sigma_r^2/\mu_r^2$) of the seed dispersal kernel from real data sets. (a) The gut-passage data for the plant-frugivore interactions in Table 2. (b) The gut-passage data for the Mediterranean avian seed dispersers in Table 3. Insets show kurtosis as a function of mean gut-passage time; this shows that similar mean retention times can generate seed dispersal curves with different degrees of kurtosis and therefore the variability in the gut-passage time scaled by its mean is needed to make reliable predictions.
